# Comparison of the HUI3 and the EQ-5D-3L in a nursing home setting

**DOI:** 10.1371/journal.pone.0172796

**Published:** 2017-02-24

**Authors:** Tom Lung, Kirsten Howard, Christopher Etherton-Beer, Moira Sim, Gill Lewin, Glenn Arendts

**Affiliations:** 1 The George Institute for Global Health, University of Sydney, Sydney, New South Wales, Australia; 2 Sydney Medical School, School of Public Health, University of Sydney, Sydney, New South Wales, Australia; 3 School of Medicine and Pharmacology Royal Perth Hospital Unit, The University of Western Australia, Crawley, Western Australia, Australia; 4 School of Medical and Health Sciences, Edith Cowan University, Joondalup, Western Australia, Australia; 5 School of Nursing, Midwifery and Paramedicine, Faculty of Health Sciences, Curtin University, Bentley, Western Australia, Australia; 6 Emergency Medicine, School of Primary, Aboriginal and Rural Health Care, The University of Western Australia, Crawley, Western Australia, Australia; National University of Singapore, SINGAPORE

## Abstract

**Background:**

Accurately assessing changes in the quality of life of older people living permanently in nursing homes is important. The multi-attribute utility instrument most commonly used and recommended to assess health-related quality of life in the nursing home population is the three-level EuroQol EQ-5D-3L. To date, there have been no studies using the Health Utilities Index Mark III (HUI3). The purpose of this study was to compare the level of agreement and sensitivity to change of the EQ-5D-3L and HUI3 in a nursing home population.

**Methods:**

EQ-5D-3L and HUI3 scores were measured as part of a cluster randomised controlled trial of nurse led care coordination in a nursing home population in Perth, Western Australia at baseline and 6-month follow up.

**Results:**

Both measures were completed for 199 residents at baseline and 177 at 6-month follow-up. Mean baseline utility scores for EQ-5D-3L (0.45; 95% CI 0.41–0.49) and HUI3 (0.15; 95% CI 0.10–0.20) were significantly different (Wilcoxon signed rank test, p<0.01) and agreement was poor to moderate between absolute scores from each instrument (intra-class correlation coefficient = 0.63). The EQ-5D-3L appeared more sensitive to change over the 6-month period.

**Conclusion:**

Our findings show that the EQ-5D-3L and HUI3 estimate different utility scores among nursing home residents. These differences should be taken into account, particularly when considering the implications of the cost-effectiveness of particular interventions and we conclude that the HUI3 is no better suited to measuring health-related quality of life in a nursing home population when compared to the EQ-5D-3L.

## Introduction

The growth in the proportion of older people [1] and those who live permanently in a nursing home requiring complex care has implications both for health service delivery and the increase in demand of expenditure in order to meet this need [2]. Health-related quality-of-life (HRQoL), measured using a multi-attribute utility instrument (MAUI), provides a summary score that estimates quality of life (QoL). These scores, in combination with survival estimates, are typically used in health economic evaluations of different interventions to estimate quality adjusted life years (QALYs) [3].

Two widely used and accepted MAUIs in economic evaluations are the Health Utilities Index Mark 3 (HUI3) and the EuroQol EQ-5D-3L [4, 5], both of which describe health states in terms of health attributes or health dimensions. A recent systematic review of instruments for measuring outcomes in economic evaluation identified a limited number of studies using MAUIs in an aged care setting [6]. All 7 studies identified used the self-reported EQ-5D-3L instrument and no studies used the HUI3 questionnaire. Four further studies used three measures which encapsulated dimensions outside of health (The Adult Social Care Outcome Toolkit (ASCOT); the ICEpop CAPability measure for Older people (ICECAP-O); and the World Health Organization Quality of Life old (WHOQOL-Old)).

Currently, the EQ-5D-3L is the recommended MAUI to use when conducting economic evaluations for health technology assessments in the UK [7] whilst for older people, in addition to the EQ-5D-3L the use of either the ICECAP-O or ASCOT is recommended in order to facilitate the measurement and valuation of both QALYs and broader quality of life dimensions of importance to the specific population [6, 8, 9]. However, there is no strong rationale for choosing the EQ-5D-3L over other MAUIs; indeed there has been concern about the EQ-5D-3L having significant limitations in older people whereby small differences in health status can result in very large changes in QOL scores [10]. Differences between the EQ-5D-3L and the HUI3 have been noted in other settings [11–13], but not in a nursing home setting.

To appropriately interpret summary HRQoL scores based on the HUI3 or EQ-5D-3L in a nursing home population, a comparison between the values and properties of these instruments is needed. In this study, we use a nursing home population from a cluster controlled clinical trial in Australian nursing homes to assess HRQOL and compare agreement and sensitivity to change between the HUI3 and EQ-5D-3L.

## Materials and methods

### Health-related quality of life measures

The EQ-5D-3L questionnaire consists of a Visual Analog Scale (VAS) and five dimensions of health: mobility, self-care, daily activities, pain/discomfort and anxiety/depression [4, 5]. Each dimension has three response levels, yielding a possible 243 unique health states. These preferences are summarised into a utility score that ranges from -0.59 to 1.0 using the time trade-off method and we use Australian derived EQ-5D-3L weights in this study [14].

The HUI3 has eight dimensions of health (vision, hearing, speech, ambulation, dexterity, emotion, cognition, and pain/complaints) and each dimension has five or six response levels, resulting in 972,000 possible unique health states. Preference-based scoring algorithms converted the descriptive health classifications into values for each health dimension [15, 16]. The health dimensions are designed to be structurally independent, and a multi-attribute model using the standard gamble method derives a utility score that ranges from -0.36 to 1.0 [16].

### Study population and data collection

Nursing home residents were recruited to a multicentre open label cluster controlled trial of nurse led care coordination in Perth, Western Australia (Australian and New Zealand Clinical Trials Registry number 12611000933954). In brief, individuals were eligible for the study if they were 65 year or older, eligible for Medicare and a permanent high level care resident in an accredited facility [17]. The EQ-5D-3L and HUI3 questionnaires were administered at baseline and 6-month follow-up, and co-morbidities were measured using the Charlson co-morbidity index [18]. Ethics approval was obtained from the University of Western Australia Human Research Ethics Committee (RA/4/1/5123). Written informed consent was obtained from all individual participants and for cognitively impaired individuals incapable of providing informed consent, provision for a written waiver of consent was granted with next of kin acknowledgement. This was directed by the supervising ethics committee and the national statement on Ethical Conduct in Human Research (‘‘National Statement on Ethical Conduct in Human Research”, 2007).

Cognitive status and disability of the resident (assessed as part of the study) determined whether they were able to self-populate the questionnaires. For individuals cognitively capable but unable (due to disability) to populate the questionnaires, a recruiter recorded resident responses at the bedside. Cognitively unable residents had a nominated proxy medical decision maker complete an informant questionnaire. Questionnaires were administered at enrolment (baseline) and at six and twelve-month follow-up. This study analyses only fully completed questionnaires at baseline and after 6 months.

For the purposes of this study, we combined both intervention and control groups in the analysis to conduct a cohort analysis of the utility scores. All analysis was conducted using Stata SE version 14.0 (StataCorp LP, College Station, TX, USA).

### Agreement between instruments

Comparisons were made at baseline between mean HUI3 and EQ-5D-3L scores by sex, number of co-morbidities, age, treatment group and method of completing the questionnaires (self-completed versus informant-completed) using the Mann-Whitney U test. The distributions of the absolute values of the two instruments were tested individually for normality (Shapiro-Wilk test) and whether the two distributions were equal using the two-sample Kolmogorov-Smirnov test. To assess agreement between the utility values from the two instruments, a Wilcoxon signed rank test was used for all sub-groups.

Kendall’s tau was computed to determine whether there was correlation between the dimensions of the EQ-5D-3L and HUI3: mobility (EQ-5D-3L) and ambulation (HUI3); pain/complaints (EQ-5D-3L) and pain (HUI3); and anxiety/depression (EQ-5D-3L) and emotion (HUI3). Correlation scores were classified into three groups: 0.1–0.3 was considered small, 0.3–0.5 as medium and >0.5 interpreted as a large correlation [19].

Intra-class correlation (ICC) estimates the proportion of variance from both instruments’ scores attributable to differences between individuals, as opposed to differences between the two instruments [20, 21]. A two-way fixed mixed effect model was computed, where the instrument effect was fixed and the individual effect was random. ICC values close to 1 suggests that the instruments are similar in terms of agreement [22].

We used Bland-Altman [23, 24] plots to graph the difference between an individual’s EQ-5D-3L and HUI3 value against the mean of the two scores, which summarises the level of agreement between the two methods.

### Sensitivity to change

The sensitivity of an instrument to changes in health was examined by calculating effect sizes. Effect size is classified as the difference between mean scores at baseline and 6-month follow-up divided by the standard deviation of baseline values. Effect sizes were defined as: small (0.20), moderate (0.50) and large (>0.80) [19]. Paired t-tests were conducted for baseline and 6-month follow-up utility values for EQ-5D-3L and HUI3, respectively to determine whether utility values changed over the observation period.

## Results

For the 200 residents in the study, 199 EQ-5D-3L and HUI3 questionnaires were completed at baseline. Residents had a mean age of 85 years and 25% were men. By six months’ follow-up, 22 residents had died and were excluded from the analysis as they did not complete a MAUI questionnaire, leaving 177. Characteristics of the study population at baseline and 6-month follow-up are reported in Table 1.

**Table 1:** Study characteristics of the population at baseline and 6-month follow-up.

Characteristics	Baseline (n = 199)	6-month follow-up (n = 177)
Age (SD*)	85.1 (8.9)	84.6 (9.0)
Male (%)	49 (24.6%)	40 (22.6%)
Female (%)	150 (75.4%)	137 (77.4%)
Charlson Comorbidity Index = 0 (%)	71 (35.7%)	63 (35.6%)
Charlson Comorbidity Index≥1 (%)	128 (64.3%)	114 (64.4%)
Control Group (%)	99 (49.7%)	90 (50.9%)
Intervention (%)	100 (50.3%)	87 (49.2%)
Informant-completed (%)	66 (33.2%)	56 (31.6%)
Self-completed (%)	133 (66.8%)	121 (68.4%)

*SD: Standard Deviation

### Agreement between instruments

The mean utility scores from both instruments measured at both baseline and 6-month follow-up are reported in Table 2. Overall, at baseline the mean scores for EQ-5D-3L and HUI3 were estimated at 0.45 (range -0.24 to 1) and 0.15 (range -0.37 to 1), respectively. Significant differences in mean utility scores were found between individuals who self-completed and those who required an informant to complete their questionnaire for both EQ-5D-3L and HUI3 scores at baseline (p<0.01) and 6 months (p<0.01). At both baseline (p<0.03) and 6-month follow-up (p<0.05), significant differences were found between the intervention and control groups for the HUI3 but not EQ-5D-3L scores.

**Table 2:** Mean utility scores and standard deviations of the study population at baseline (n = 199) and 6-month (n = 177) follow-up for EQ-5D-3L and Health Utilities Index Mark 3 (HUI3).

Characteristics	Mean baseline scores (SD)		Mean 6 month scores (SD)	
	EQ-5D-3L	HUI3	EQ-5D-3L	HUI3
Overall	0.45 (0.02)	0.15 (0.02)	0.44 (0.02)	0.16 (0.03)
Age<85 years	0.44 (0.03)	0.15 (0.04)	0.41 (0.03)	0.15 (0.04)
Age≥85 years	0.46 (0.03)	0.15 (0.03)	0.46 (0.03)	0.17 (0.03)
Male	0.45 (0.05)	0.17 (0.05)	0.44 (0.05)	0.18 (0.05)
Female	0.45 (0.03)	0.14 (0.03)	0.44 (0.03)	0.15 (0.03)
Charlson Comorbidity Index = 0	0.45 (0.04)	0.14 (0.04)	0.45 (0.04)	0.15 (0.04)
Charlson Comorbidity Index>0	0.45 (0.03)	0.16 (0.03)	0.43 (0.03)	0.17 (0.03)
Control Group	0.44 (0.03)	0.09* (0.03)	0.44 (0.03)	0.10* (0.03)
Intervention	0.46 (0.03)	0.21* (0.04)	0.44 (0.04)	0.21* (0.04)
Informant-completed	0.28* (0.03)	-0.05* (0.03)	0.25* (0.04)	-0.04* (0.03)
Self-completed	0.53* (0.03)	0.25*(0.03)	0.53* (0.03)	0.25* (0.03)

*Represents a significant (p<0.05) Mann-Whitney test which rejects the null hypothesis that mean utility scores of sub-groups are from populations with the same distribution.

At baseline, the EQ-5D-3L distribution shows positive skewness whereas the HUI3 distribution shows negative skewness (S1 Fig). The assumption of normality for both utility scores (Shapiro-Wilk test) and equality of the two distributions (Two-sample Kolmogorov-Smirnov test) was strongly rejected (p<0.01). The distribution of scores at 6 months are similar to those at baseline (S2 Fig). Wilcoxon signed rank tests strongly rejected the null hypothesis of equality between individual’s EQ-5D-3L and HUI3 utility scores at both baseline and 6 months’ follow-up in all sub-groups listed in Table 1. At baseline, the majority of HUI3 scores (87%) were lower than the corresponding EQ-5D-3L score.

Large positive correlations (S1 Table) were found between the dimensions Pain/Complaints (EQ-5D-3L) and Pain (HUI3) (Kendall’s tau = 0.51), moderately positive correlation between mobility (EQ-5D-3L) and ambulation (HUI3) (Kendall’s tau = 0.40) and a small positive correlation was found between anxiety/depression (EQ-5D-3L) and Emotion (HUI3). All correlations were strongly statistically significant (p<0.01).

**Fig 1:**
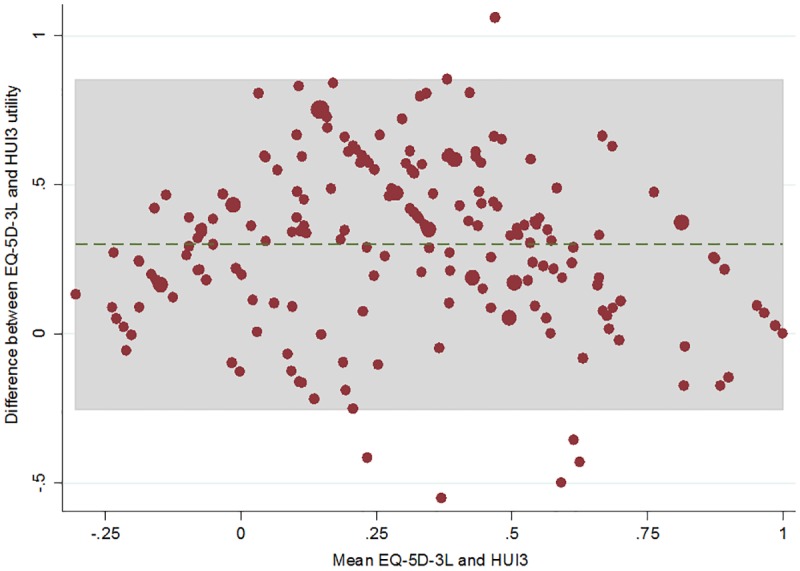
Bland-Altman plot of mean EQ-5D-3L versus HUI3 utility values and the difference between the two utility values at baseline (n = 199).

The ICC coefficient was 0.63 (95% CI 0.54–0.71), implying a poor to moderate level of agreement between the two instruments (S2 Table). Fig 1 depicts a Bland-Altman plot at baseline, showing a mean difference of 0.3 between EQ-5D-3L and HUI3 scores. The scatterplot shows that as mean utility values increase, the difference of values between the two instruments decreases.

### Sensitivity to change

Effect sizes between baseline and 6-month follow-up for EQ-5D-3L (0.19) and HUI3 (0.02) suggested that the EQ-5D-3L was more sensitive than HUI3 to changes over a 6-month follow-up. Paired t-tests between baseline and 6 months’ follow-up strongly rejected the null hypothesis that the mean difference between EQ-5D-3L scores was greater than zero (p<0.02), suggesting a deterioration of EQ-5D-3L utility values over the observation period. No statistically significant differences were found between baseline and 6-month follow-up HUI3 values.

## Discussion

This is the first study that has compared two commonly used MAUIs, the EQ-5D-3L and HUI3, in a nursing home setting. We investigated agreement and sensitivity to change for utility scores based on the EQ-5D-3L and HUI3, providing insight into the differences between these two instruments for people living in a nursing home.

At the individual level, the intra-class correlation coefficients and the Bland-Altman plot reveals a poor to moderate level of agreement between the two instruments, however moderate and small positive correlations exist between some health dimensions. EQ-5D-3L utility scores were considerably higher than corresponding HUI3 scores, which corroborates with non-nursing home population literature [11–13]. The multiplicative scoring function of the HUI3 assumes that all dimensions are dependent on one another, a possible explanation of the lower utility values estimated by the HUI3 in this nursing home cohort where polymorbidity is common.

Only EQ-5D-3L scores showed a statistically significant difference between baseline and follow-up. One reason for the larger deterioration in utility values when using the EQ-5D-3L is the smaller number of health states available, so a slight change in one health dimension from no or moderate impairment at baseline to severe impairment at follow-up could result in a relatively large decrement in health utility values.

The nursing home population poses considerable challenges for researchers using MAUIs. The two main areas of difficulty are a necessity to use proxy/informant measures of QoL in a large proportion of the population and the applicability of instruments for a population that, by definition, has lost the capacity to live independently in the community. When compared to self-completed scores, informant ratings of QoL are consistently lower in nursing home [25] and elderly community dwellings [26, 27], findings replicated in this study for both MAUIs. It is important to stress that broader elements of QoL (i.e. privacy, dignity, autonomy, etc.) [28, 29] as defined by elderly nursing home residents should be measured and valued alongside a MAUI in order to capture all potential benefits of an intervention. Compared to the seven studies that used EQ-5D-3L scores in an aged care setting [6], we found a similar baseline mean EQ-5D-3L score of 0.45 (SD = 0.32) and within the 95% confidence interval of all but one of the reported studies [30–33]. As this is the first study that used the HUI3 in a nursing home setting, future comparison and validation must be conducted to determine whether similar utility values are estimated.

A strength of this study was the 100% completion rate for those alive at 6-month follow-up for both MAUIs, which enabled an unbiased examination of change in HRQoL scores and their change over time. A limitation of the present study was our inability to examine whether there were differences in utility values according to the order in which instruments were administered. In all subjects, the EQ-5D-3L was administered first, followed by the HUI3. Changes over time in questionnaire administration mode (i.e. self-completion at baseline to proxy/informant at 6-month follow-up or vice versa) were not captured, which may have influenced the variation in mean scores between baseline and 6-month follow-up. We did not measure cognitive burden of the two questionnaires, which can be an important consideration in MAUI assessment in older people. The HUI3 contains up to 41 questions, which is substantially longer than the EQ-5D-3L (5 questions). One study comparing the Assessment of Quality of Life (AQoL) (15 questions) to the EQ-5D-3L in an aged population found that participants were more burdened by the AQoL and consequently found it more difficult to complete [10]. Unlike the EQ-5D-3L, the HUI3 is not based upon preferences of the Australian general population, which could account for some differences seen between the instruments in our results. However, the HUI3 has been widely applied in Australian contexts and appears to behave consistently with other multi-attribute utility instruments, despite the non-Australian based scoring system [34–37].

Overall, this study has demonstrated that the EQ-5D-3L appears to be more sensitive to changes over a 6-month period compared to the HUI3, yet there appears to be a low level of agreement between the two. Due to the potential cognitive burden of the HUI3, we conclude that the HUI3 appears no better suited to measuring HRQoL in a nursing home setting than the EQ-5D-3L. Given the increasing use of the EQ-5D-5L (with 5 response levels instead of three level in each QOL domain), additional confirmatory studies may be needed.

## Conclusions

In conclusion, there were significant differences when comparing EQ-5D-3L and HUI3 scores in a nursing home population. Over a 6-month period the EQ-5D-3L appears to be more sensitive to changes in health status. Overall, the HUI3 appears no better suited than the EQ-5D-3L in this context. Further studies are required to determine the appropriateness of the HUI3 in this population, particularly given the cognitive burden when compared to the EQ-5D-3L.

## Supporting information

S1 Fig Distribution of EQ-5D-3L and HUI3 scores at baseline. (TIF)

S2 Fig Distribution of EQ-5D-3L and HUI3 scores at 6-month follow-up. (TIF)

S3 Fig Bland-Altman plot of mean EQ-5D-3L versus HUI3 utility values and the difference between the two utility values at 6-month follow-up (n = 177). (TIF)

S1 Table Kendall’s tau to determine the agreement between the health state descriptions. (DOCX)

S2 Table Intra-class coefficient two-way fixed effects model estimating the level of consistency between the two instruments. (DOCX)
