# Supplementary figures and images for: Comparison of the HUI3 and the EQ-5D-3L in a nursing home setting
Source: PLoS One. 2017 Feb 24;12(2):e0172796. doi: 10.1371/journal.pone.0172796 (PMC5325524; doi:10.1371/journal.pone.0172796)

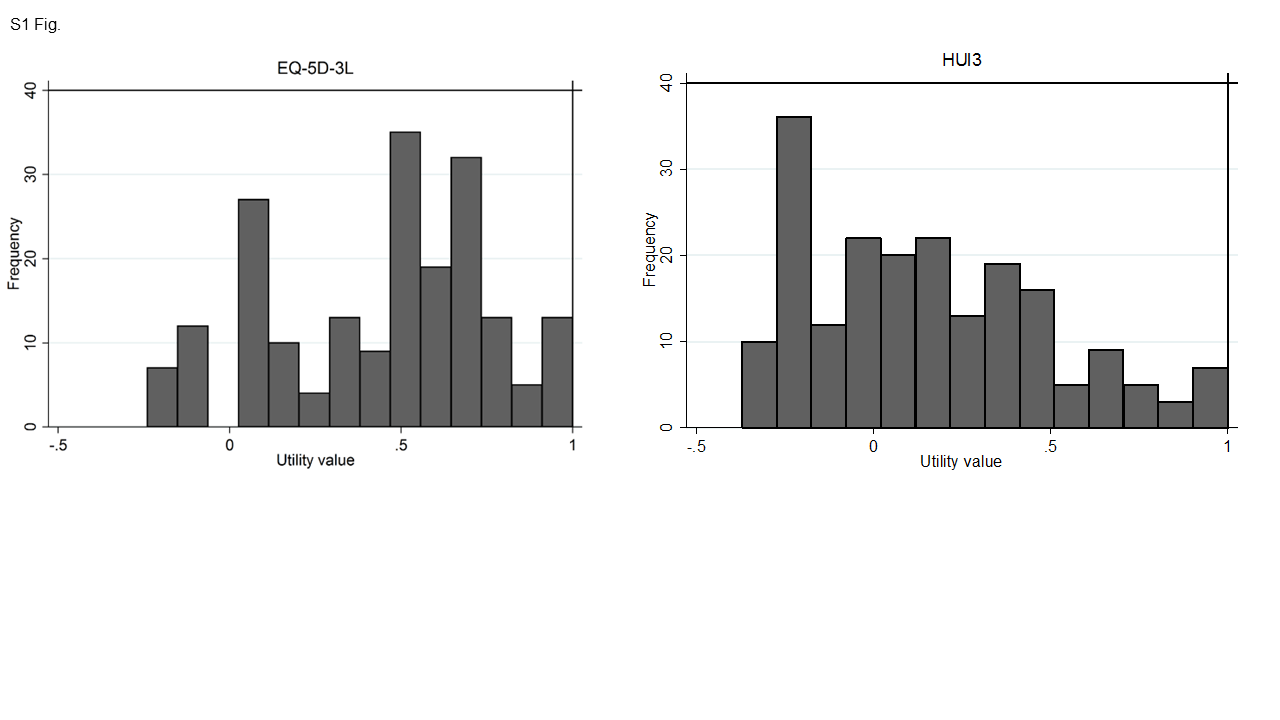

Supplement: S1 Fig — (TIF) [file pone.0172796.s001.tif]

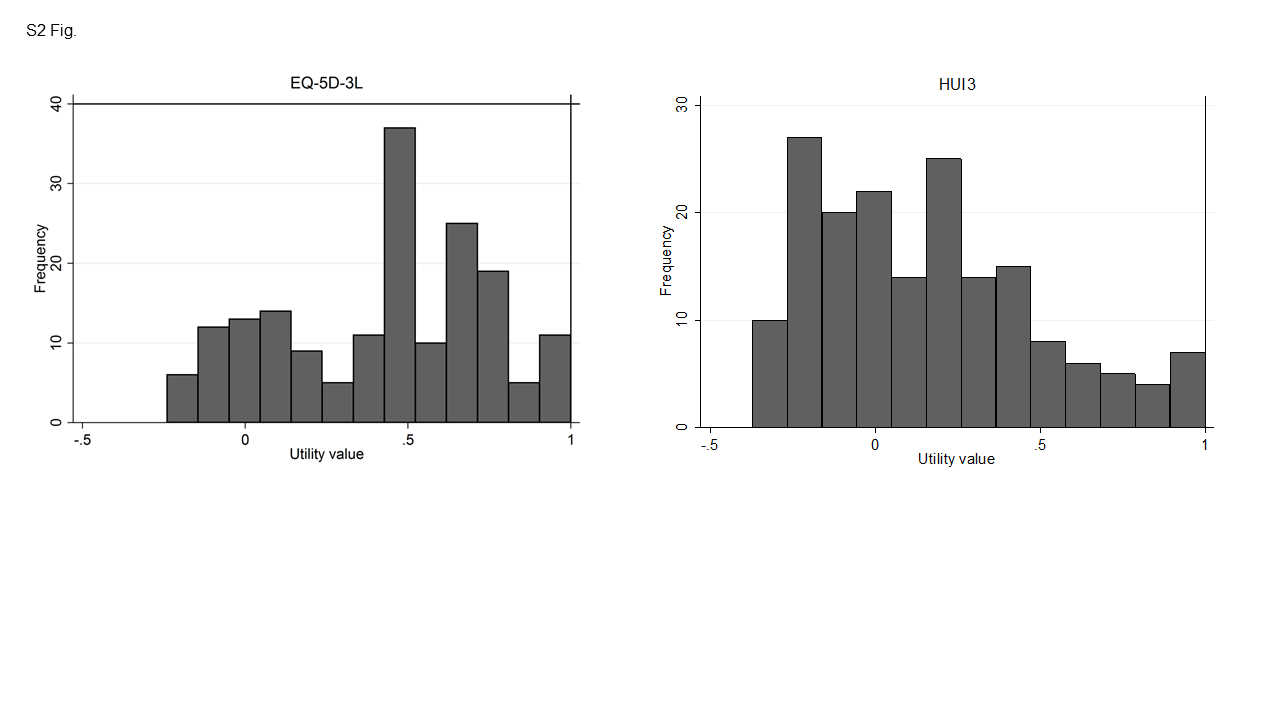

Supplement: S2 Fig — (TIF) [file pone.0172796.s002.tif]

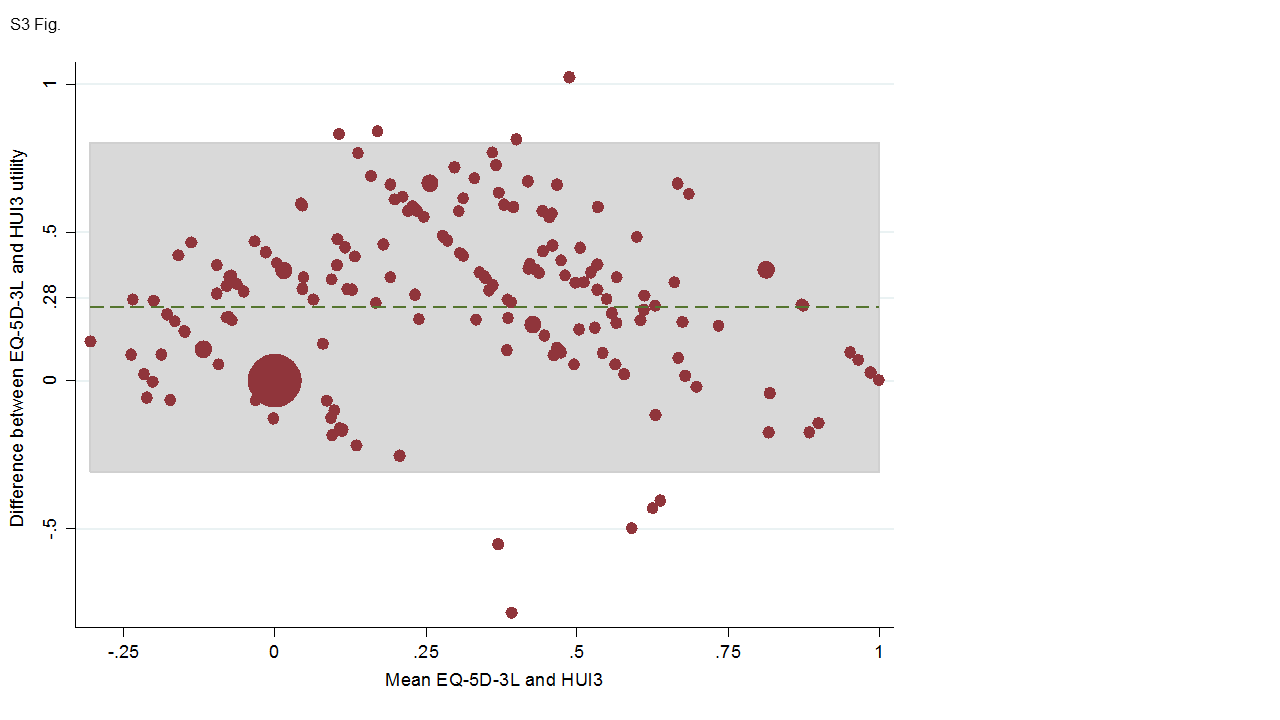

Supplement: S3 Fig — (TIF) [file pone.0172796.s003.tif]
