# Supplementary material for: Comparison of the HUI3 and the EQ-5D-3L in a nursing home setting
Source: PLoS One. 2017 Feb 24;12(2):e0172796. doi: 10.1371/journal.pone.0172796 (PMC5325524; doi:10.1371/journal.pone.0172796)
Supplement: S1 Table — (DOCX) [file pone.0172796.s004.docx]

S1 Table - Kendall’s tau to determine the agreement between the health state descriptions

| EQ-5D-3L dimension | HUI3 Dimension | Kendall’s tau statistic |
| --- | --- | --- |
| Mobility | Ambulation | 0.40 (p<0.01) |
| Pain/Complaints | Pain | 0.51 (p<0.01) |
| Anxiety/Depression | Emotion | 0.20 (p<0.01) |
