# Supplementary material for: Comparison of the HUI3 and the EQ-5D-3L in a nursing home setting
Source: PLoS One. 2017 Feb 24;12(2):e0172796. doi: 10.1371/journal.pone.0172796 (PMC5325524; doi:10.1371/journal.pone.0172796)
Supplement: S2 Table — (DOCX) [file pone.0172796.s005.docx]

S2 Table - Intra-class coefficient two-way fixed effects model estimating the level of consistency between the two instruments

| Baseline utility | Intra-class coefficient | 95% Confidence Interval |
| --- | --- | --- |
| Individual | 0.63 | (0.54 – 0.71) |
| Average | 0.77 | (0.70 – 0.83) |
